# Psychosocial factors associated with smoking and drinking among Japanese early adolescent boys and girls: Cross-sectional study

**DOI:** 10.1186/1751-0759-1-13

**Published:** 2007-07-04

**Authors:** Mikayo Ando, Takashi Asakura, Shinichiro Ando, Bruce G Simons-Morton

**Affiliations:** 1 Department of Counseling and Educational Psychology, Center for Research and Development in Education, Okayama University, 3-1-1 Tsushimanaka, Okayama 700-8530, Japan; 2 Health and Social Behavior Research Laboratory, Faculty of Education, Tokyo Gakugei University, 4-1-1 Nukuikita-machi, Koganei, Tokyo 184-8501, Japan; 3 Department of Internal Medicine, Okayama Citizen's Hospital, 6-10 Amase, Okayama 700-8557, Japan; 4 Prevention Research Branch, Division of Epidemiology Statistics and Prevention Research, National Institute of Child Health and Human Development, National Institutes of Health, 6100 Executive Boulevard, 7B05, Bethesda, Maryland 20892-7510, USA

## Abstract

**Background:**

Smoking and drinking alcohol among early adolescents are serious public health concerns, but few studies have been conducted in Japan to assess their prevalence and etiology. A regional survey was conducted in eight schools in two Japanese school districts to identify psychosocial factors associated with smoking and drinking behaviors for boys and girls.

**Methods:**

Junior high school students from seventh to ninth grades (N = 2,923) completed a self-reported questionnaire between December 2002 and March 2003. Relationships between psychosocial variables (i.e., self-assertive efficacy to resist peer pressure, parental involvement, school adjustment, and deviant peer influence) and smoking and drinking were investigated using logistic regression analyses and path analyses.

**Results:**

Smoking in the last six months was significantly more prevalent in boys (7.9%) than girls (5.1%). The prevalence of drinking in the last six months was similar in boys (23.7%) and girls (21.8%). Self-efficacy to resist peer pressure was negatively associated with both smoking and drinking among both boys and girls and provided both direct and indirect effects through deviant peer influence. Parental involvement showed indirect effects through school adjustment and/or deviant peer influence to both smoking among both boys and girls and drinking among girls, although parental involvement showed direct effects on smoking only for boys. School adjustment was negatively associated with smoking among both boys and girls and drinking among girls.

**Conclusion:**

These findings suggest that self-assertive efficacy to resist peer pressure, parental involvement, school adjustment and deviant peer influence are potentially important factors that could be addressed by programs to prevent smoking and/or drinking among early adolescent boys and girls in Japan.

## Background

Adolescent smoking and drinking are serious national concerns in Japan [1]. Early experimentation with tobacco and alcohol is associated with both immediate and lasting threats to healthful youth development, including abuse and dependence, which can result in long-term health and social consequences [2].

Among Japanese junior high school students, 58.2–76.0% reported having tried alcohol and 17.7–45.0% reported having tried tobacco [3-5]. Smoking (41.8%) and drinking (3.4%) have also been listed as causes for protective police custody of teenagers (ages 14 to 19) [1]. Factors associated with adolescent smoking and drinking have not been well researched in Japan, although some studies reported associations between lower family-related self-esteem and smoking and drinking among Japanese early adolescents [5,6].

Social cognitive theory [7] and problem behavior theory [8] contribute to our understanding as to why adolescents begin to smoke and drink. According to social cognitive theory, behavior continually interacts with personal factors such as self-efficacy, as well as family and school environments. For instance, peer influence can occur when youths associate with peers who smoke or drink alcohol, resulting in an increase in availability, providing role models, establishing smoking and drinking as normative, and creating the perception that using them might increase social acceptance [7,9]. According to problem behavior theory, experimentation with smoking and drinking is likely to bring adolescents into contact with other youths who smoke and drink alcohol and engage in other problem behaviors.

A variety of factors has been found to be associated with adolescent smoking and drinking. High parent expectations, involvement, and monitoring have been found to be negatively associated with use [10-12]. Peer influence is another factor commonly linked to adolescent smoking and drinking [11-15]. The influence of school variables, such as social support, adjustment, and positive climate are also important in protecting youth from participating in smoking and drinking [16-18]. However, it is unclear how these variables may interact in their adolescent involvement in smoking and drinking.

Furthermore, limited information is available as to how influences on smoking and drinking may differ for male and female adolescents. High perceived prevalence of smoking, positive attitudes toward smoking, deviance acceptance, trouble at school, and negative family relationships have been found to be associated with smoking for both boys and girls [11,19]. In other research, peer influence, less authoritative parenting, low mastery, and low social conformity were associated with smoking for boys, whereas self-control problems, low parental involvement, less social support from family and friends, and parental smoking were associated with smoking only for girls [11,20,21]. In other research, high-perceived prevalence of drinking, deviance acceptance, low self-control [22], and high drinking expectation with low parental expectations against problem behaviors [23] were associated with drinking for both boys and girls. Grade point average [22] was negatively associated with drinking for boys, whereas propensity for risk-taking [22] and peer pressure were associated with drinking for girls [12]. Peer influence was associated with drinking for both boys and girls, but more so for girls than boys [12].

Importantly, these risk factors for adolescent smoking and drinking have been studied in countries of western culture, but not in Japan. Japanese people have some unique characteristics, such as interdependent orientation and collective cultural thinking, which stresses conformity and intense competition for sameness within intragroup relations [24,25]. These characteristics may influence the prevalence and etiology of adolescent smoking and drinking.

The purpose of this study is to assess how psychosocial variables influence smoking and drinking and how these associations may be common or unique for each gender among Japanese early adolescents. Based on the social cognitive and problem behavior theories and the literature review, we used path analyses to examine the associations between self-efficacy, parent involvement, school adjustment, and peer influence and smoking and drinking among Japanese early adolescent boys and girls to explore factors that could be targeted by interventions designed to prevent smoking and drinking for youths.

## Methods

### Participants

A survey was administered to 7th–9th grade students (12–15 years of age) in eight public junior high schools in two suburban cities located within 150 km from the center of Tokyo between December 2002 and March 2003. Of 3,486 students eligible to participate in this survey, 2,923 (83.8%) completed the survey (1,478 boys and 1,445 girls; 885 7th graders, 1,016 8th graders, and 1,022 9th graders).

### Procedure

School consent was obtained from each school principal. In their home-base classrooms, students were asked by teachers for their assent to participate. The teachers administered and proctored the survey questionnaire. To ensure privacy, the students were not required to identify themselves by name or identification number. The study was reviewed and approved by the United Graduate School of Education Tokyo Gakugei University Institutional Review Board and authorized by the Board of Education in each city.

### Measures

The study used a self-reported questionnaire concerning tobacco and alcohol use and psychosocial variables. Some items, selected from scales written in English, were translated into Japanese. Considering the cultural differences, the measures were modified based on a pilot study with junior high school students. Each psychosocial variable included in the survey was analysed using the sum of the scores of the scale items were supported by the confirmatory factor analysis [26]. These variables are described on Table 1.

**Table 1:** Independent variables, number of items, Cronbach' α, and item

**Variable**	**No. of items**	**Possible range of scores**	**Cronbach' α**	**Items**
Self-assertive efficacy to resist peer pressure				
Self-assertive efficacy against smoking	1	1–5	Item	How much assertiveness can you express when you are asked by your friends about smoking? (cannot express, cannot express somewhat, neither of them, can express a little, can express)
Self-assertive efficacy against drinking	1	1–5	Item	How much assertiveness can you express when you are asked by your friends about drinking? (cannot express, cannot express somewhat, neither of them, can express a little, can express)
Parental involvement	5	5–25	0.81	How much do your parents or guardians know about your friends; activities; health habits; how you spend your time after school and on weekends; how you are doing at school? (almost nothing, little, neither know nor unknown, a little, a lot)
School adjustment	3	3–15	0.73	How hard or easy is it for you to... follow rules, pay attention in class, and get along with teachers? (much harder, a little harder, neither harder nor easier, a little easier, much easier)
Deviant peer influence				
Number of friends who smoke	1	0–4	Item	How many of your four closest friends (0–4) smoke?
Number of friends who drink alcohol	1	0–4	Item	How many of your four closest friends (0–4) drink alcohol?

Psychosocial variables, number of items, internal consistency of index, and items with response options are shown.

Tobacco and alcohol use: The assessment of smoking and drinking was based on measures used in previous adolescent health studies [1,27,28]. Students were asked about their use of tobacco and alcohol in the last six months. Response options ranged between 0 = "none", 1 = "one or two times", 2 = "sometimes", 3 = "once a week", or 4 = "more than two or three times a week". Each behavior was analysed independently.

Self-assertive efficacy to resist peer pressure on smoking or drinking: Self-assertive efficacy to resist peer pressure was assessed by items developed by Caprara et al [29]. Respondents were asked, "How much assertiveness can you express when you are asked by your friends about smoking/drinking?" The items were rated on a 5-point scale (1 = "cannot express", 2 = "cannot express somewhat", 3 = "neither of them", 4 = "can express a little", 5 = "can express").

Parental involvement: Parental involvement was assessed by the short-form Parent Involvement Scale [30], originally developed by Hetherington and Clingempeel [31]. Assessment involved how much the respondents thought their parents/guardians knew about their friends and school life. The items were rated on a 5-point scale (1 = "almost nothing", 2 = "little", 3 = "neither know nor unknown", 4 = "a little", 5 = "a lot").

School adjustment: School adjustment was assessed with items from the School Adjustment Scale [32]. Students rated how hard or easy the items described their school lives. The items were rated on a 5-point scale (1 = "much harder", 2 = "a little harder", 3 = "neither harder nor easier", 4 = "a little easier", 5 = "much easier").

Deviant peer influence: Deviant peer influence was assessed by the Deviant Peer Influence Scale [32]. It asked, "How many of your four closest friends use tobacco/alcohol?" The items were rated on a 5-point scale (0 = "none", 1 = "one friend", 2 = "two friends", 3 = "three friends", 4 = "four friends").

### Statistical analyses

Smoking and drinking in the last six months served as the dependent variables for all analyses. Prevalence was calculated and compared by gender using the Mann-Whitney U test as two-independent-sample tests and the Kruskal Wallis test as multiple comparison tests. Due to the low prevalence of smoking and drinking among early adolescents, the dependent variables were dichotomized into no use or any use in the last six months.

Results were analysed by means of SPSS 14.0J for Windows [33]. First, bivariate correlations for all variables were performed. Then, the simple and multiple logistic regression analyses examined the influence of the psychosocial variables on smoking and drinking separately for each gender. In the dependent variables, no use of tobacco or alcohol was used as the referent. In the independent variables, the smallest value in each range of the scores was used as the referent.

Finally, variables were further examined in path analyses. Based on theory and the results of previous research, variables were treated either as predictor or mediator variables in the path analyses. School variables (deviant peer influence and school adjustment) were treated as hypothesized mediators [14,34]. Separate path analyses were performed for each of the four groups (smoking or drinking, and boys and girls). To identify the adequacy of the path models, several restricted models were first conducted based on the multiple logistic regression analyses and the significances of the indirect, direct and total effects using the bias-corrected confidence intervals of bootstrapping [35-37]. Model estimations were conducted with Amos 7.0 [38]. Model fit was assessed with the root mean square error of approximation (RMSEA), the comparative fit index (CFI), and the Tucker-Lewis index (TLI) [37], which are better than typical chi-square goodness-of-fit measures for large samples, [36,39]. Generally, a RMSEA value less than 0.05 indicates a good fit; a RMSEA less than 0.08 indicates an adequate fit between the hypothesized model and the observed data [37,40]. The model is better when CFI and TLI are near to 1.0 [37]. The level of significance was set to *p *< 0.05.

## Results

### Prevalence of smoking and drinking

The prevalence of smoking and drinking are shown in Table 2 and Table 3, respectively.

**Table 2:** Prevalence of smoking by grade

	**None**		**1–2 times**		**Sometimes**		**Once a week**		**More than 2–3 times a week**		
	***N***	**(%)**	***N***	**(%)**	***N***	**(%)**	***N***	**(%)**	***N***	**(%)**	
Total^a^	2713	(93.5)	84	(2.9)	66	(2.3)	7	(0.2)	32	(1.1)	
Boys	1350	(92.1)	47	(3.2)	41	(2.8)	4	(0.3)	24	(1.6)	^**b^
Girls	1363	(94.9)	37	(2.6)	25	(1.7)	3	(0.2)	8	(0.6)	
Grade^a^											^**d^
7th	843	(95.9)	18	(2.0)	15	(1.7)	2	(0.2)	1	(0.1)	
Boys	426	(95.7)	9	(2.0)	8	(1.8)	1	(0.2)	1	(0.2)	
Girls	417	(96.1)	9	(2.1)	7	(1.6)	1	(0.2)	0	(0)	
8th	935	(92.9)	35	(3.5)	21	(2.1)	4	(0.4)	12	(1.2)	^**e^
Boys	449	(90.5)	20	(4.0)	14	(2.8)	3	(0.6)	10	(2.0)	^*c^
Girls	486	(95.1)	15	(2.9)	7	(1.4)	1	(0.2)	2	(0.4)	
9th	935	(92.0)	31	(3.1)	30	(3.0)	1	(0.1)	19	(1.9)	^***f^
Boys	475	(90.5)	18	(3.4)	19	(3.6)	0	(0)	13	(2.5)	
Girls	460	(93.7)	13	(2.6)	11	(2.2)	1	(0.2)	6	(1.2)	

Prevalence of tobacco use in the last six months among Japanese adolescent boys and girls by grade are shown.

Significant at * *p *< 0.05; ** *p *< 0.01; *** *p *< 0.001 (two-tailed).

^a ^Does not total 2,923 due to missing data. (*N *= 2,902)

^b ^In the two-independent-sample tests by the Mann-Whitney U test among gender, the prevalence among boys was significantly higher than among girls.

^c ^In 8th graders, the prevalence among boys was significantly higher than among girls.

^d ^In the multiple comparison tests by the Kruskal Wallis test, there was significant difference among grades.

^e ^In the two-independent-sample tests by the Mann-Whitney U test between each of the two grades, the prevalence among 8th graders was higher than among 7th graders.

^f ^The prevalence among 9th graders was higher than among 7th graders.

**Table 3:** Prevalence of drinking by grade

	**None**		**1–2 times**		**Sometimes**		**Once a week**		**More than 2–3 times a week**		
	***N***	**(%)**	***N***	**(%)**	***N***	**(%)**	***N***	**(%)**	***N***	**(%)**	
Total^a^	2241	(77.2)	324	(11.2)	245	(8.4)	42	(1.4)	50	(1.7)	
Boys	1118	(76.3)	158	(10.8)	130	(8.9)	21	(1.4)	39	(2.7)	
Girls	1123	(78.2)	166	(11.6)	115	(8.0)	21	(1.5)	11	(0.8)	
Grade^a^											^**b^
7^th^	708	(80.5)	81	(9.2)	68	(7.7)	14	(1.6)	8	(0.9)	^***c^
Boys	356	(80.0)	38	(8.5)	36	(8.1)	7	(1.6)	8	(1.8)	
Girls	352	(81.1)	43	(9.9)	32	(7.4)	7	(1.6)	0	(0)	
8^th^	786	(78.1)	111	(11.0)	80	(7.9)	14	(1.4)	16	(1.6)	^*d^
Boys	377	(76.0)	55	(11.1)	46	(9.3)	6	(1.2)	12	(2.4)	
Girls	409	(80.0)	56	(11.0)	34	(6.7)	8	(1.6)	4	(0.8)	
9^th^	747	(73.5)	132	(13.0)	97	(9.5)	14	(1.4)	26	(2.6)	
Boys	385	(73.3)	65	(12.4)	48	(9.1)	8	(1.5)	19	(3.6)	
Girls	362	(73.7)	37	(13.6)	49	(10.0)	6	(1.2)	7	(1.4)	

Prevalence of alcohol use in the last six months among Japanese adolescent boys and girls by grade are shown.

Significant at * *p *< 0.05; ** *p *< 0.01; *** *p *< 0.001 (two-tailed).

^a ^Does not total 2,923 due to missing data. (*N *= 2,902)

^b ^In the multiple comparison tests by the Kruskal Wallis test, there was significant difference among grades.

^c ^In the two-independent-sample tests by the Mann-Whitney U test between each of the two grades, the prevalence among 9th graders was higher than among 7th graders.

^d ^The prevalence among 9th grader was higher than among 8th graders.

The prevalence of smoking was significantly higher among boys than girls (*p *< 0.01). In eighth graders, the prevalence of smoking was significantly higher among boys than girls (*p *< 0.05). In the analyses by the Kruskal Wallis test, the prevalence of smoking by grade was significantly different (*p *< 0.01). In the analyses between each of the two grades by the Mann-Whitney U test, the prevalence of smoking among 8th and 9th graders were higher than among 7th graders (*p *< 0.01, *p *< 0.001, respectively).

In the analyses by the Kruskal Wallis test, the prevalence of drinking among grades was significantly different (*p *< 0.01). In the analyses between each of the two grades by the Mann-Whitney U test, the prevalence of drinking among 9th graders was higher than among 7th and 8th graders (*p *< 0.001, *p *< 0.05, respectively).

### Logistic regression analyses

Assessment of the bivariate correlations for all variables separately for each gender revealed no significant multicollinearity.

Tables 4 and 5 show the odds ratios and 95% confidence intervals for the psychosocial characteristics and last six month smoking and drinking separately by gender. In the simple logistic regression analyses (Table 4), all psychosocial variables were significant for both smoking and drinking among both boys and girls. The number of friends who smoke or drink alcohol was highly and positively association with smoking and drinking for both boys and girls.

**Table 4:** Simple logistic regression analyses

	**Tobacco use**				**Alcohol use**			
	
	**Boys (*N *= 1,402)**		**Girls (*N *= 1,396)**		**Boys (*N *= 1,402)**		**Girls (*N *= 1,396)**	
	
**Variable**	**OR^a^**	**95%CI^b^**	**OR^a^**	**95%CI^b^**	**OR^a^**	**95%CI^b^**	**OR^a^**	**95%CI^b^**
**Self-assertive efficacy against smoking or drinking**	0.43	0.36 – 0.47	0.38	0.32 – 0.46	0.52	0.47 – 0.57	0.42	0.37 – 0.47
**Parental involvement**	0.85	0.82 – 0.89	0.88	0.84 – 0.93	0.94	0.91 – 0.97	0.91	0.88 – 0.94
**School adjustment**	0.78	0.74 – 0.84	0.76	0.70 – 0.82	0.88	0.84 – 0.91	0.83	0.79 – 0.87
**Number of friends who smoke or drink alcohol**	2.71	2.30 – 3.19	3.11	2.45 – 3.94	2.12	1.87 – 2.40	2.79	2.35 – 3.30

Odds ratios and 95% confidence intervals for psychosocial variables influencing tobacco and alcohol use among Japanese boys and girls by simple logistic regression analyses are shown.

^a ^Odds ratio. ^b ^Confidence interval.

Bold indicates significant association to the dependent variables at *p *< 0.05 (two-tailed).

**Table 5:** Multiple logistic regression analyses

	**Tobacco use**				**Alcohol use**			
	
	**Boys (*N *= 1,402)**		**Girls (*N *= 1,396)**		**Boys (*N *= 1,402)**		**Girls (*N *= 1,396)**	
	
**Variable**	**OR^a^**	**95%CI^b^**	**OR^a^**	**95%CI^b^**	**OR^a^**	**95%CI^b^**	**OR^a^**	**95%CI^b^**
Self-assertive efficacy against smoking or drinking	**0.50**	**0.43 – 0.59**	**0.44**	**0.36 – 0.54**	**0.60**	**0.54 – 0.66**	**0.51**	**0.44 – 0.57**
Parental involvement	**0.92**	**0.88 – 0.97**	0.97	0.91 – 1.04	1.00	0.96 – 1.03	0.99	0.95 – 1.03
School adjustment	**0.90**	**0.83 – 0.97**	**0.82**	**0.74 – 0.91**	0.97	0.92 – 1.02	**0.90**	**0.85 – 0.95**
Number of friends who smoke or drink alcohol	**2.19**	**1.83 – 2.63**	**2.39**	**1.82 – 3.14**	**1.74**	**1.53 – 1.98**	**2.11**	**1.77 – 2.52**

Odds ratios and 95% confidence intervals for psychosocial variables influencing tobacco and alcohol use among Japanese boys and girls by multiple logistic regression analyses are shown.

^a ^Odds ratio. ^b ^Confidence interval.

Bold indicates significant association to the dependent variables at *p *< 0.05 (two-tailed).

In the multiple logistic regression analyses (Table 5), self-assertive efficacy against smoking or drinking and number of friends who smoke or drink alcohol were significant for both smoking and drinking among both boys and girls. In addition, school adjustment was significantly associated with smoking among both boys and girls and drinking among girls. Parental involvement was only significantly associated with smoking among boys.

### Path Analyses

**Figure 1:**
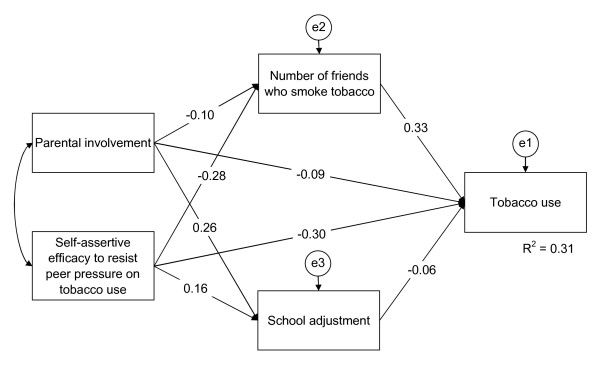
**Path model of smoking structure for boys**. The final path model of smoking structure for boys, χ^2 ^(d.f. = 1, *N *= 1,402) = 7.17, *p *= 0.007; CFI = .0.993, TLI = 0.933, and RMSEA = 0.066. Straight single-headed arrows indicate standardized path coefficients. e1, e2, and e3 indicate unmeasured errors associated with each of the variables in the model. Double-headed arrow indicates covariance of exogenous variable. All variables show significant and direct effects to tobacco use at *p *< .05. Self-assertive efficacy to resist peer pressure and parental involvement indicate significant indirect effects to tobacco use through the mediators, number of friends who smoke tobacco and school adjustment, at *p *< .05.

**Figure 2:**
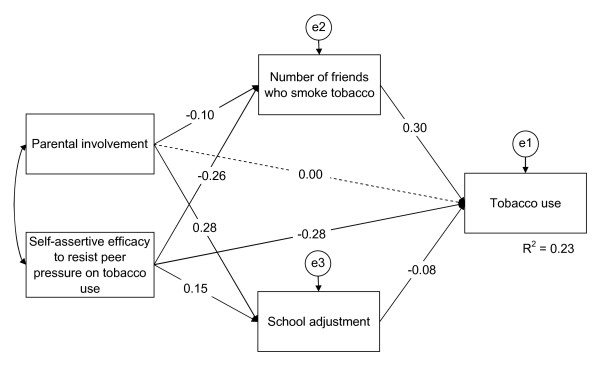
**Path model of smoking structure for girls**. The final path model of smoking structure for girls, χ^2 ^(d.f. = 2, *N *= 1,396) = 15.00, *p *= 0.001; CFI = .0.982, TLI = 0.910, and RMSEA = 0.068. Dashed single-headed arrows mean that the regression weights were restricted as zero. Straight single-headed arrows indicate standardized path coefficients. e1, e2, and e3 indicate unmeasured errors associated with each of the variables in the model. Double-headed arrow indicates covariance of exogenous variable. Variables with straight single-headed arrows indicate significant and direct effects to tobacco use at *p *< .05. Self-assertive efficacy to resist peer pressure and parental involvement indicate significant indirect effects to tobacco use through the mediators, number of friends who smoke tobacco and school adjustment, at *p *< .05.

**Figure 3:**
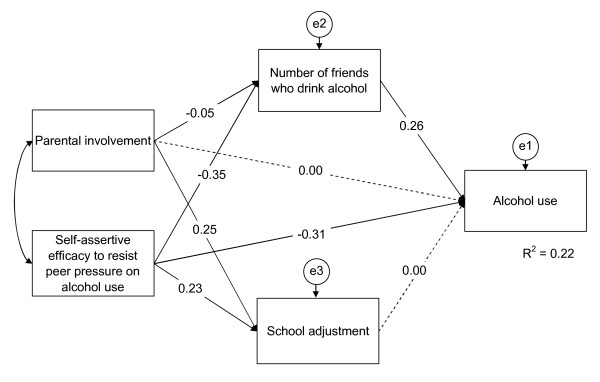
**Path model of drinking structure for boys**. The final path model of drinking structure for boys, χ^2 ^(d.f. = 3, *N *= 1,402) = 10.90, *p *= 0.012; CFI = 0.991, TLI = 0.969, and RMSEA = 0.043. Dashed single-headed arrows mean that the regression weights were restricted as zero. Straight single-headed arrows indicate standardized path coefficients. e1, e2, and e3 indicate unmeasured errors associated with each of the variables in the model. Double-headed arrow indicates covariance of exogenous variable. Variables with straight single-headed arrows indicate significant and direct effects to alcohol use at *p *< .05. Self-assertive efficacy to resist peer pressure shows significant indirect effects through a mediator, number of friends who drink alcohol, at *p *< .05.

**Figure 4:**
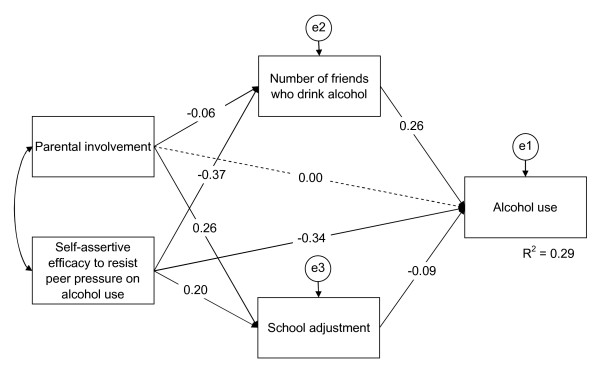
**Path model of drinking structure for girls**. The final path model of drinking structure for girls, χ^2 ^(d.f. = 2, *N *= 1,396) = 6.20, p = 0.045; CFI = 0.996, TLI = 0.978, and RMSEA = 0.039. Dashed single-headed arrows mean that the regression weights were restricted as zero. Straight single-headed arrows indicate standardized path coefficients. e1, e2, and e3 indicate unmeasured errors associated with each of the variables in the model. Double-headed arrow indicates covariance of exogenous variable. Variables with straight single-headed arrows indicate significant and direct effects to alcohol use at *p *< .05. Self-assertive efficacy to resist peer pressure and parental involvement indicate significant indirect effects to alcohol use through the mediators, number of friends who drink alcohol and school adjustment, at *p *< .05.

The best fitting path model for tobacco and alcohol use and gender are shown in Figures 1, 2, 3, 4.

Smoking among boys: Shown in Figure 1 are eight of the standardized path coefficients that were significant at *p *< 0.05 for the smoking among the boys' path model. This model fit the data adequately, χ^2 ^(d.f. = 1, *N *= 1,402) = 7.17, *p *= 0.007 (CFI = 0.993; TLI = 0.933; RMSEA = 0.066). Self-assertive efficacy to resist peer pressure on smoking, parental involvement, school adjustment, and number of friends who smoke were significantly associated with smoking in the direct paths. Parental involvement and self-assertive efficacy to resist peer pressure on smoking were significantly associated with smoking in the indirect paths through the variables, school adjustment and number of friends who smoke.

Smoking among girls: Shown in Figure 2 are seven of the standardized path coefficients that were significant at *p *< 0.05 for the smoking among the girls' path model. This model fit the data adequately, χ^2 ^(d.f. = 2, *N *= 1,396) = 15.00, *p *= 0.001 (CFI = 0.982; TLI = 0.910; RMSEA = 0.068). Self-assertive efficacy to resist peer pressure on smoking, school adjustment, and number of friends who smoke were significantly associated with smoking in the direct paths. Self-assertive efficacy to resist peer pressure on smoking and parental involvement was significantly associated with smoking in the indirect path through the variables, school adjustment and number of friends who smoke.

Drinking among boys: Shown in Figure 3 are six of the standardized path coefficients that were significant at *p *< 0.05 for the drinking among the boys' path model. This model fit the data well, χ^2 ^(d.f. = 3, *N *= 1,402) = 10.90, *p *= 0.012 (CFI = 0.991; TLI = 0.969; RMSEA = 0.043). Self-assertive efficacy to resist peer pressure on drinking and number of friends who drink were significantly associated with drinking in the direct paths. Self-assertive efficacy to resist peer pressure on drinking was significantly associated with drinking in the indirect path through the number of friends who drink.

Drinking among girls: Shown in Figure 4 are seven of the standardized path coefficients that were significant at *p *< 0.05 for the drinking among the girls' path model. This model fit the data well, χ^2 ^(d.f. = 2, *N *= 1,396) = 6.20, *p *= 0.045 (CFI = 0.996; TLI = 0.978; RMSEA = 0.039). Self-assertive efficacy to resist peer pressure on drinking, school adjustment, and number of friends who drink were significantly associated with drinking in the direct paths. Self-assertive efficacy to resist peer pressure on drinking was significantly associated with drinking in the indirect path through the variables, school adjustment and number of friends who drink. Parental involvement was also significantly associated with drinking in the indirect path through the variable, school adjustment.

Then, the path models for smoking and drinking were combined as path models for substance use by including common variables associated with them by gender. The models were not found to fit the data adequately, for boys χ^2 ^(d.f. = 9, *N *= 1,402) = 467.78, *p *< 0.001 (CFI = 0.868; TLI = 0.691; RMSEA = 0.191) and for girls χ^2 ^(d.f. = 13, *N *= 1,396) = 500.92, *p *< 0.001 (CFI = 0.850; TLI = 0.677; RMSEA = 0.164).

## Discussion

This study investigated cross-sectional association with smoking and drinking in a regional sample of Japanese junior high school students to determine possible differences and similarities of psychosocial factors associated directly with or indirectly through school variables between boys and girls. This is one of few studies to examine the etiology of adolescent smoking and drinking in Japan. It is necessary to identify modifiable factors associated with tobacco and alcohol use that can be targeted in intervention programs.

The prevalence of smoking and drinking were similar to those of other reports in Japan [1,3]. In our sample, smoking was significantly more prevalent in higher grades, and more prevalent in boys. Drinking was significantly more prevalent in higher grades, but similar among boys and girls, as has generally been found to be the case [12].

From the path analyses, deviant peer affiliation and low self-assertive efficacy against smoking and drinking were the most important and consistent risk factors for tobacco and alcohol use among both boys and girls. Deviant peer affiliation also mediated the relationship between self-assertive efficacy and tobacco and alcohol use among boys and girls commonly. School adjustment was associated with tobacco use for both boys and girls and alcohol use was associated for girls but not for boys directly. School adjustment also mediated the relationships between parental involvement and self-efficacy and tobacco use for both boys and girls and alcohol use for girls but not for boys. It is interesting that parental involvement showed indirect effects through school adjustment and/or deviant peer affiliation to smoking for both boys and girls and drinking for girls but not for boys. Furthermore, parental involvement showed direct effects on smoking prevalence only for boys. These variables have all been found to be important in US studies as well.

The relationship between tobacco and alcohol use and association with deviant peers may be due to selection or socialization [34]. That is, adolescents who are interested in using tobacco and alcohol may select friends with similar interests or may be influenced to use tobacco and alcohol by associating with friends who use them. Consequently, adolescents often behave in similar ways as their close friends. The interactions between self-efficacy and association with tobacco and alcohol through friends indicate that self-efficacy is mediated by peer affiliation. Possibly, adolescents' self-efficacy may be relatively more protective depending on the attitudes and behaviors of their close friends. Students who formed a positive affiliation or social bond with their schools and schoolmates, who liked their classes, who believed their teachers to be supportive and fair, and who had good peer relationships, and who accepted their school's mission, values, and standards, were less likely to engage in problem behaviors [11].

In the Japanese collective culture, group activities and group goals are emphasized. Japanese adolescents may want to be part of the group at all costs. [24]. Therefore, deviant peer influence may be a particularly important factor associated with smoking and drinking. Self-assertion in maintaining good interpersonal relationships may be a particularly important skill among Japanese youth for resisting peer pressure concerning smoking and drinking in a collective cultural context.

The findings from this study suggest that peer influence and self-assertive efficacy to resist peer pressure are potentially important targets for intervention to prevent both smoking and drinking for both male and female junior high school students in Japan.

As peer influence was a partial mediator between the predictor and outcome variables, school-based intervention program designed to improve school engagement, adjustment, and commitment and increase pro-social friendships might effectively protect against tobacco and alcohol initiation and progression. Parental involvement is another potentially important factor to consider in developing preventive interventions. Although a number of national health objectives address the prevention of smoking and drinking, effective preventive interventions have not been fully developed. Most interventions to date have focused on increasing knowledge about substance use, perceived consequences of their use, and anti-drug use attitudes (information-dissemination approaches), but evaluations have provided little evidence of effects on behavior [41,42].

We are currently implementing in junior high schools a psychoeducational program designed to increase self-control, school adjustment, adequate friendships, and self-efficacy to prevent problem behaviors including tobacco and alcohol use [43]. Evaluation of a pilot program showed some positive changes related with smoking and drinking [44]. Based on the current results, we will expand the current psychoeducational program to enhance capabilities to handle conflict and to resist peer pressure using scenarios related to smoking and drinking. Based on the findings of the current study, there is a clear need to extend intervention to facilitate parental involvement.

In Japanese society, adults often use drinking as an important factor for successful business. University/college students often drink and meet as part of school club activities. These cultures might influence the behavior of early adolescents. Nozu [45] argued that intervention to prevent drinking should impact this cultural background and he recommends approaches to improve resiliency.

It is important to acknowledge several limitations of the present study. All information collected in the present study was gathered from self-reported questionnaires. Although multiple procedures were used to ensure anonymity, it is possible that some respondents chose to provide socially desirable answers. The rate of participants who did not answer the questions on smoking and drinking was less than 0.7%, quite good for surveys; however, 16.2% of the students refused to participate or were absent. The non-participants may have been more likely to smoke and drink than students who participated, thereby providing an underestimate of the true prevalence. Such underreporting presents a conservative bias serving mainly to reduce the magnitude of associations. The direction of relationships cannot be determined in cross-sectional research. Therefore, the path analyses provide speculative views of the hypothesized relationships only. Prospective research is needed to determine the causal pathways between psychosocial factors and tobacco and alcohol use.

## Conclusion

Path analyses of data from a regional survey of 7th–9th graders in eight Japanese schools found that deviant peer influence, self-assertive efficacy to resist peer pressure, parental involvement, and school adjustment are important factors related with smoking and/or drinking among early adolescent boys and girls in Japan.

## Competing interests

The author(s) declare that they have no competing interests.

## Authors' contributions

All authors read and approved the final manuscript. MA conceptualised and designed the study, coordinated the translation process, collected and analysed the data, interpreted the results and drafted the manuscript. TA supervised the analysis, and helped to revise the manuscript. SA supported the translation and interpretation, and helped to draft and revised the manuscript. BGS supervised the analysis and interpretation, and helped to draft and revise the manuscript.
